# How to detect and correct myocardial creep in myocardial perfusion imaging using Rubidium-82 PET?

**DOI:** 10.1007/s12350-019-01650-x

**Published:** 2019-02-20

**Authors:** S. S. Koenders, J. D. van Dijk, P. L. Jager, J. P. Ottervanger, C. H. Slump, J. A. van Dalen

**Affiliations:** 1 Department of Nuclear Medicine, Isala hospital, PO Box 10400, 8000 GK Zwolle, The Netherlands; 2 Department of Medical Physics, Isala hospital, Zwolle, The Netherlands; 3 Department of Cardiology, Isala hospital, Zwolle, The Netherlands; 4 MIRA: Institute for Biomedical Technology and Technical Medicine, University of Twente, Enschede, The Netherlands

**Keywords:** Myocardial blood flow, PET myocardial perfusion imaging, ^82^Rb, myocardial creep, pharmacological vasodilators

## Abstract

Reliability of myocardial blood flow (MBF) quantification in myocardial perfusion imaging (MPI) using PET can majorly be affected by the occurrence of myocardial creep when using pharmacologically induced stress. In this paper, we provide instructions on how to detect and correct for myocardial creep. For example, in each time frame of the PET images the myocardium contour and the observed activity have to be compared to check for misalignments. In addition, we provide an overview of the functionality of commonly used software packages to perform this quality control step as not all software packages currently provide this functionality. Furthermore, important clinical considerations to obtain accurate MBF measurements are given.

## Introduction

**Figure 1:**
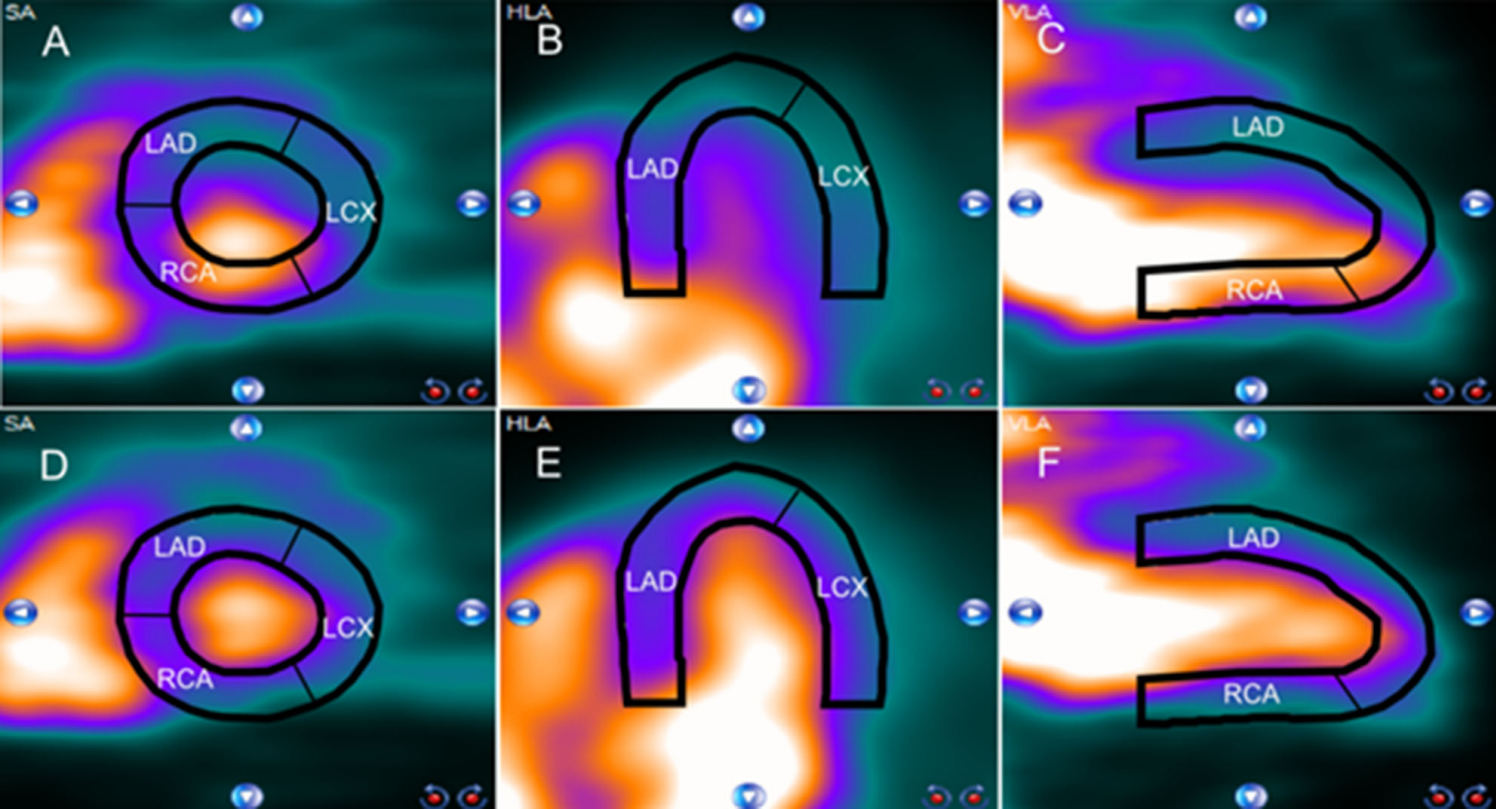
Example of a stress Rb-82 PET scan of a patient with myocardial creep, before (**A** to **C**) and after myocardial creep correction (**D** to **F**). The myocardium contour is shown in black and the vascular trajectories that primarily supply certain areas of the myocardium with blood are indicated. The appearance of myocardial creep is indicated by the misalignment between the observed Rb-82 activity and the myocardium contour (**A** to **C**). Especially the activity concentration in the right coronary artery (RCA) territory is affected when comparing the uncorrected (**A** to **C**) with the corrected images (**D** to **F**). From left to right: the short axis, horizontal axis, and vertical long axis. *LAD*, left anterior descending; *LCX*, left circumflex artery

Myocardial blood flow (MBF) quantification in myocardial perfusion imaging (MPI) using Rubidium-82 (Rb-82) PET provides valuable information about the extent and functional importance of possible stenosis.^1^–^3^ However, the reliability of MBF quantification can be affected by the occurrence of myocardial creep, in particular during stress imaging.^4^ This myocardial creep is presumably caused by the increasing respiration and lung volume and thereby repositioning of the diaphragm and heart after administration of a pharmacological vasodilator.^5^,^6^ It mainly affects activity concentration measurements in the right coronary artery (RCA) territory as illustrated in Figure 1.^4^ As activity concentration measurements are used in compartmental analyses to derive MBFs, it is essential that these measurements are reliable to prevent biased MBF measurements and thereby false diagnostic interpretation.^4^

In our recent study, we observed a myocardial creep during regadenoson-induced stress in 52% of the 104 consecutively included patients.^4^ In 83% of these 54 patients, myocardial creep resulted in a MBF change > 10%, which may influence diagnostic interpretation. Although our study only comprised regadenoson-induced stress, the presence of myocardial creep is also reported with adenosine as pharmacological vasodilator.^6^ In a limited amount of patients (2%), myocardial creep can also affect MBF quantification using rest imaging.^4^ As MBF quantification can become biased when myocardial creep remains uncorrected, detection and correction are necessary for all pharmacological vasodilators and for both rest and stress scans. In this paper, we show how myocardial creep can be detected and corrected. Furthermore, we provide an overview of the possibilities of commercially available software packages to detect and correct myocardial creep and highlight important clinical considerations.

## Methodology

### Background: MBF Quantification

Several steps have to be performed prior to quantification of MBF: (1) dynamic PET acquisition; (2) image reconstruction of the PET data; (3) segmentation of the myocardium contour; (4) derivation of time-activity curves (TACs) of the myocardium and the left ventricle (LV); (5) quality control; and (6) compartmental analyses.^7^

The first step starts with a PET acquisition of typically 7 minutes for both the rest and stress scans directly after Rb-82 administration. Typically, a low-dose CT scan is added to provide an attenuation map of the chest to allow attenuation correction. Next, the PET images are reconstructed in several time frames (step 2) where the first-pass phase or blood-pool phase is generally sampled with small frame durations of five to ten seconds to assure sufficient temporal resolution and prevent under-sampling of the LV TAC.^8^–^10^

Subsequently, a myocardium contour is drawn, based on all data acquired during the tissue phase where a steady state is reached, i.e., data acquired >2:15 minutes after Rb-82 administration (step 3),^11^ as the activity is then primarily present in the myocardium. This contour is used to derive the activity concentrations over time for the whole myocardium or a specific myocardial region. The most common regions are those supplied by blood by one of the three main coronary arteries: left anterior descending (LAD), left circumflex (LCX), and RCA. In addition, the activity concentration in the LV is estimated by using, for example, a region of interest (ROI) positioned in the cavity of the LV. Both the myocardium contour and the LV ROI are used to automatically derive TACs (step 4). To calculate the MBF for the whole myocardium or a specific region, the TACs from the corresponding myocardial area and the LV are used as input for compartmental analyses. The one-tissue compartment model is most commonly used for this analysis when using Rb-82 (step 6).^8^

To obtain reliable MBF measurements, a quality control (step 5) has to be performed which covers the detection and correction of myocardial creep. We previously defined myocardial creep as a gradual decreasing misalignment of the myocardium contour with the activity present in the ventricle and/or myocardium primarily in the inferior direction.^4^ Myocardial creep should be corrected if the misalignment is more than one-third of the width of the left ventricular myocardial wall and is present in at least 2 time frames during the first-pass phase.^4^

## Myocardial Creep Detection and Correction

As it is essential to check and correct for myocardial creep,^4^ we first provide instructions for detection and correction in general, followed by an example based on commercial processing software (Corridor4DM, Invia).

### General Procedure

**Figure 2:**
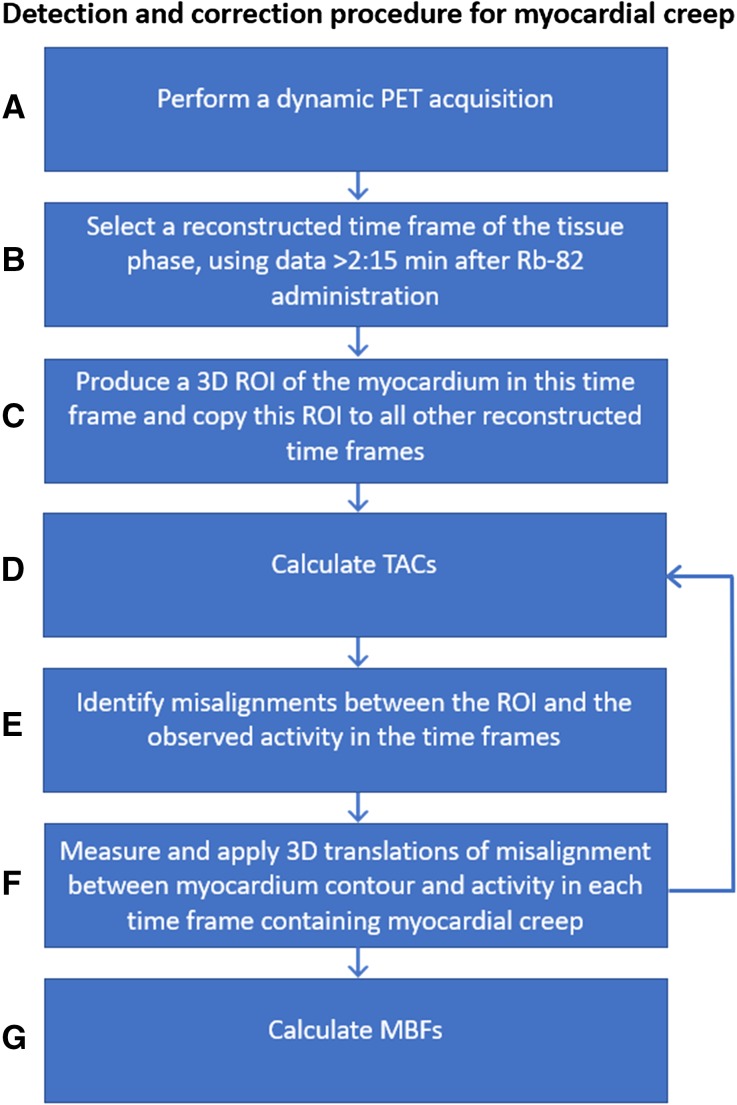
General procedure for the detection and correction of myocardial creep

The detection and correction procedure consists of seven steps, as shown in Figure 2A to G. After the PET data are acquired (A), the geometric position of the myocardium contour has to be determined (B) to detect myocardial creep. This is generally done by reconstructing the PET data collected after 2:15 minutes into one image, as the activity is then primarily present in the myocardium. It is important that this image reconstruction is based on a sufficient number of photon counts to provide a clear image of the myocardium. Next, the geometric position of the myocardium can be obtained by drawing a 3D ROI with a fixed threshold of typically 70% of the maximum pixel value in the myocardium (C). The myocardium contour then needs to be copied to all the other time frames of the dynamic acquisition. After the TACs are calculated (D), the position of the 3D ROI and the observed activity distribution in each frame have to be compared (E) as misalignment may indicate myocardial creep.

If myocardial creep is present, it can be corrected for by estimating the misalignment in the x-, y- and z-direction for each time frame in which myocardial creep is visible (F). This geometrical translation can be used to realign the observed activity to the myocardium contour by, for example, changing the initial coordinates in the DICOM header of the PET data for each of the time frames containing myocardial creep. The calculation of the TACs then has to be repeated to calculate reliable MBFs (G).

### Illustration Using Commercial Software

**Figure 3:**
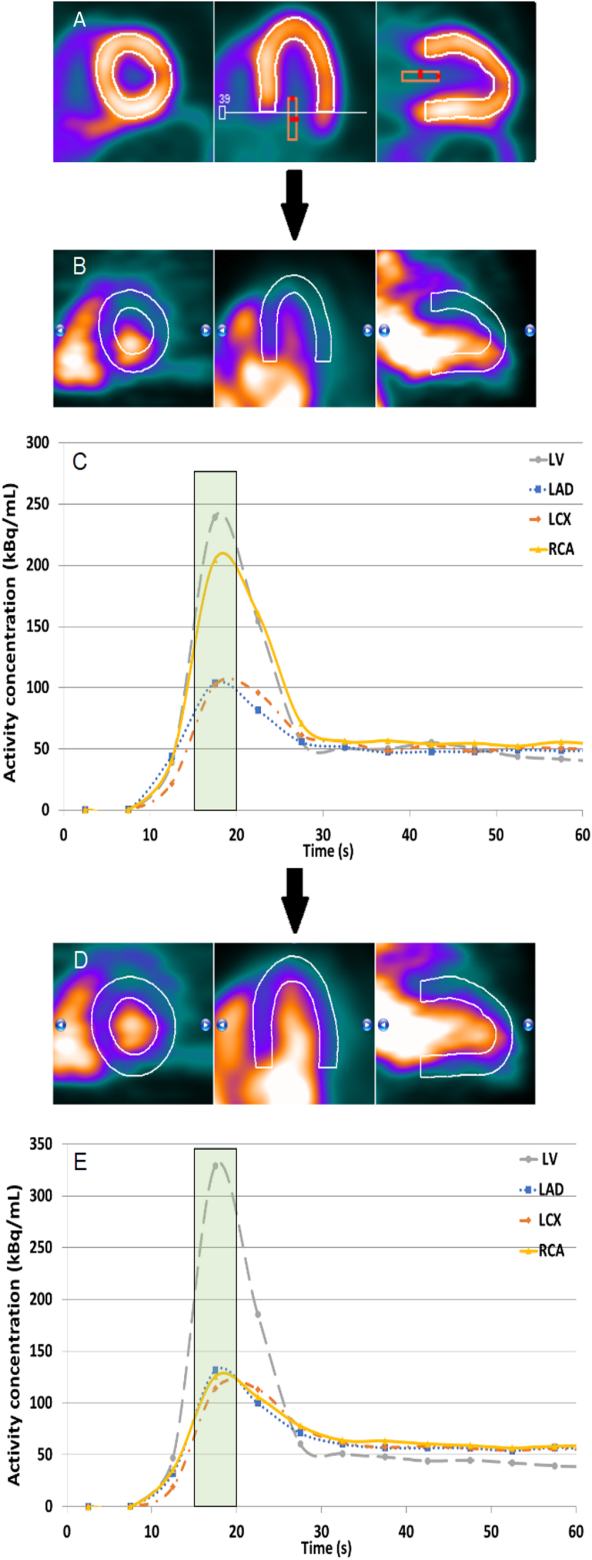
Overview of the three main steps to detect and correct for myocardial creep using Corridor4DM. The myocardium contour is drawn by assigning the most basal part of the septum which still contains activity, and the activity concentration in the left ventricle (LV) is measured by placing a region of interest (ROI) manually at the center of the mitral valve (**A**). To detect myocardial creep, the observed activity in the myocardium has to be compared visually with the myocardium contour in each time frame. The misalignment in the time frame from 15 to 20 seconds shown in **B** indicates myocardial creep. The first 60 seconds of the TAC of this time frame (**C**) shows a higher peak in the right coronary artery (RCA) territory compared to those of the other two vascular territories, indicating myocardial creep. In **D**, the observed activity in the myocardium is realigned to the myocardium contour. This results in comparable peaks of the TACs of the three vascular territories (**E**). From left to right (**A**, **B**, **D**): the short axis, horizontal axis, and vertical long axis. *LAD*, left anterior descending; *LCX*, left circumflex artery

It is possible to perform the detection and correction steps in some commercially available software, for example in Corridor4DM v2016. This software automatically derives an image reconstruction of the acquired PET data between 2:30 and 6:00 minutes after Rb-82 administration. After assigning the three cardiac axes, a myocardium contour is automatically drawn in the PET image which can manually be optimized if needed. Next, the user has to manually position a ROI at the center of the mitral valve. This ROI is used to estimate the activity concentration in the LV, as illustrated in Figure 3A. The myocardium contour is then automatically projected to all time frames of the dynamic PET series.

Corridor4DM has the option to scroll through the time frames which makes it possible to detect myocardial creep, as shown in Figure 3B. Myocardial creep can also be identified by observing the TACs. The TAC of the RCA territory then typically shows a higher peak during the first-pass phase compared to those of the other territories (Figure 3C). This higher peak is due to motion of the heart in the inferior direction, which is related to myocardial creep.

Besides detecting myocardial creep, Corridor4DM also provides the possibility to correct for this movement by manually realigning the myocardium contour with the activity for each individual time frame, as shown in Figure 3D. After applying this manual realignment in each time frame with myocardial creep, the peaks of the TACs of the three vascular territories (LAD, LCX, and RCA) become comparable (Figure 3E). This ensures the user that a reliable correction for myocardial creep is performed, allowing reliable MBF measurements.

## Availability in Commercial Software Packages

As myocardial creep may hamper diagnostic interpretation, accurate detection and correction of myocardial creep are necessary for reliable MBF quantification. Although the detection is most of the time straightforward, correction can be complicated and is not always feasible in the clinical routine due to missing functionality of the used software. From the latest versions of four commonly known and used commercially software packages to quantify MBF using Rb-82 PET, Corridor4DM and QPET (Cedars-Sinai) have the ability to visually evaluate the detection and correction of myocardial creep. SyngoMBF (Siemens Healthcare) provides the functionality to automatically detect and correct for motion, such as myocardial creep, but does not provide insight in the accuracy of the correction. Moreover, it is not possible to manually adjust this correction. Lastly, FlowQuant (University of Ottawa Heart Institute) currently does not have a feature for detection and correction of myocardial creep.

## Considerations

Measurements of MBF using Rb-82 PET are affected by many methodological factors such as differences in equipment, acquisition and reconstruction settings, processing software, tracer infusion, temporal sampling, and compartmental analyses.^12^ Awareness of all potential pitfalls and underlying assumptions in methodology are essential for using MBF measurements in clinical practice. For example, it is important that a constant activity injection profile is used together with an adequate number and length of time frames, to prevent under-sampling and that myocardial creep is adequately corrected.

Although we focused on Rb-82 PET, it is likely that myocardial creep occurs in a similar way using other PET tracers such as Oxygen-15 water and Nitrogen-13 ammonia. Therefore, detection and correction should always be performed in quantitative PET MPI studies, independent of the tracer.

Physicians should always check for accurate myocardial creep correction before clinical interpretation. This can be performed by inspecting the TAC for an elevated peak of the RCA during the first-pass phase in comparison to the LAD and LCX as shown in Figure 3C.^4^ Physicians can also visually assess the individual time frames for misalignments between the myocardium contour and the activity in the myocardium as shown in Figure 3B.

In conclusion, adequate detection and correction of myocardial creep are crucial for reliable MBF quantification. To adequately perform the required quality control, it is not only important that software packages provide the possibility to detect and correct myocardial creep, but also that users can visually inspect and evaluate these steps. Hence, vendors should provide this functionality or adapt their software accordingly.

## Abbreviations

CT: Computed tomography
LAD: Left anterior descending
LCX: Left circumflex
MBF: Myocardial blood flow
MPI: Myocardial perfusion imaging
PET: Positron emission tomography
Rb-82: Rubidium-82
RCA: Right coronary artery
TAC: Time-activity curve
